# Capacity-building during public health emergencies: perceived usefulness and cost savings of an online training on SARS-CoV-2 real-time polymerase chain reaction (qPCR) diagnostics in low- and middle-income settings during the COVID-19 pandemic

**DOI:** 10.3389/fpubh.2024.1197729

**Published:** 2024-06-07

**Authors:** Heide Weishaar, Francisco Pozo-Martin, Brogan Geurts, Estibaliz Lopez de Abechuco, Eloisa Montt-Maray, Florin Cristea, Seth Kofi Abrokwa, Thurid Bahr, Sameh Al-Awlaqi, Charbel El Bcheraoui

**Affiliations:** Evidence-Based Public Health Unit, Centre for International Health Protection, Robert Koch Institute, Berlin, Germany

**Keywords:** COVID-19, diagnostics, online training, usefulness, cost

## Abstract

**Introduction:**

Upon the onset of the COVID-19 pandemic, the Public Health Laboratory Support Unit (ZIG4) at the Robert Koch Institute (RKI), the German National Public Health Institute, developed and delivered an online training on SARS-CoV-2 qPCR diagnostics to 17 partner countries in low- and middle-income countries (LMIC). This article analyses the usefulness and cost savings of this training.

**Methods:**

The authors performed a concurrent mixed-methodology study based on key informant interviews, interviewer-administered questionnaires, and document reviews. Economic costs were estimated from the perspective of RKI.

**Results:**

Responding participants indicated that the course provided good and comprehensive information on up-to-date scientific knowledge and laboratory practice in PCR diagnostics. Respondents appreciated how the technical content of the training enhanced their ability to apply diagnostic methods in their daily work. Interviewees highlighted that the fast implementation and the low threshold of attending an online training had allowed them to quickly build skills that were crucial during, and beyond, the COVID-19 crisis. The total estimated cost of the online SARS-CoV-2 qPCR training was 61,644 euros. The total estimated cost of the equivalent face-to-face training was estimated at 267,592 euros. Programme weaknesses identified included the top-down approaches taken, lack of interactive components and opportunities to directly engage with other course participants and with teachers.

**Conclusions:**

An online training was developed and implemented to support RKI partner countries in SARS-CoV-2 qPCR diagnostics during the COVID-19 pandemic, thereby strengthening pandemic response and health system resilience. The training incurred in important cost savings compared to the equivalent face-to-face training. Post-pandemic studies could usefully build on these research findings and explore ways to enhance end user involvement and improve interactive features to build stronger communities of learners and facilitate exchange of information and mutual learning.

## Introduction

The COVID-19 pandemic has demonstrated the limitations of health systems worldwide and revealed that many health systems lack resilience (1). Particular problems have been encountered as traditional ways of operating, e.g., through face-to-face interactions, could not be followed. An important task of health systems during a public health emergency is to control the chains of transmission (2). In order to prepare and respond to public health emergencies, countries need to prioritize the implementation of efficient diagnostic strategies and improved diagnostic capacities (3). During the COVID-19 pandemic, countries had to quickly react to establish and optimize diagnostic strategies and capacities in order to adapt to the constantly changing evidence and the epidemiological situation. In low- and middle-income countries (LMIC), where adequate surveillance, laboratory capacities and resources to perform diagnostic testing had been lacking even prior to the pandemic, this challenge was even more pronounced (3, 4). Health systems in LMIC often rely on international donors and governments to complement and capacitate their national health systems in order to ensure that the necessary equipment and logistics are in place for adequate diagnostics, such as by providing diagnostic kits, reagents and laboratory equipment (3, 5). In addition to bringing about logistical challenges, diagnostic systems require a high level of technical expertise (6). Yet, capacity building in this area requires sufficient funding as well as access to relevant events, networks and trainings, including regional or international conferences, online and offline training programs and courses, professional meetings, and laboratory and academic networks. Such resources are even more crucial as new technologies are introduced, and laboratory expertise needs to be constantly updated and personnel needs to be trained on new diagnostic procedures. Unfortunately, the necessary funds and access to appropriate training, networks and events are often scarce in LMIC (7–9) which means that the necessary capacities to perform diagnostics remain often limited to a handful of experts. While these obstacles have been recognized for more than a decade (5) and substantial progress has been made regarding surveillance and preparedness for outbreaks since the West Africa Ebola pandemic, the COVID-19 pandemic has once again highlighted weaknesses across health systems and demonstrated a need to invest in building the capacity of laboratory personnel as a means to prepare for and respond to future outbreaks. Despite recent efforts, more capacity-building and training is still required to address the challenges in responding to public health and health emergencies in LMIC especially with regard to the development needs for the daily operation of public health laboratories (10).

While face-to-face trainings are still the format that is used most in laboratory training, existing studies suggest that online digital tools can be an effective way to acquire laboratory skills, especially when combined with on-site teaching in a hybrid or blended learning model (11). Given the rapid spread of COVID-19 and the travel restrictions imposed, face-to-face training that involved traveling and face-to-face interactions had to be canceled and training was limited to online delivery. Even though online trainings have shown to work well with regard to knowledge-building through delivering content and an understanding of relevant laboratory processes, they have also shown to have limitations with regard to the development of practical laboratory skills, e.g., familiarizing students with equipment, techniques and materials or developing diagnostic skills (12). Additional challenges in distance education have been identified regarding the limited feedback and interaction between students and supervisors and the unstable internet connectivity in LMIC (12).

Existing studies on online delivery of laboratory practices highlight that online training must be carefully designed to cover the various aspects of laboratory work, from the experimental design and analytical skills to the development of the technical judgement (12, 13). However, to the best of or knowledge, there is no data available on the overall cost savings associated with online compared to face-to-face diagnostics laboratory trainings. In addition, there is a gap in knowledge regarding the perceived benefits and disadvantages that such trainings might have. Even less is known about the applicability and suitability of online laboratory trainings in public health emergencies.

## Online SARS-CoV-2 qPCR diagnostic training

In February of 2020, i.e., immediately upon onset of the COVID-19 pandemic, the Public Health Laboratory Support Unit (ZIG4) of the Center for International Health Protection (ZIG) at the Robert Koch Institute (RKI), the German National Public Health Institute, received funding from the German Federal Ministry of Health to provide a training on SARS-CoV-2 qPCR diagnostics to partner countries. Initially, the training was planned to be delivered in three distinct African sites, each targeting a number of neighboring countries. During the first stages of the training program, and with the evolving COVID-19 situation, travel restrictions were imposed, prohibiting ZIG4 scientists from reaching the training sites and delivering the training. With the new situation, ZIG4 modified the training to be delivered online. The online training was disseminated through several of RKI's partner organizations that had close links to laboratories, organizations and potential beneficiaries of the training in the targeted regions, including Africa Centers for Disease Control and Prevention (ACDC) and WHO regional offices for the African (WHO AFRO), European (WHO EURO), and Eastern Mediterranean (WHO EMRO) regions.

**Figure 1:**
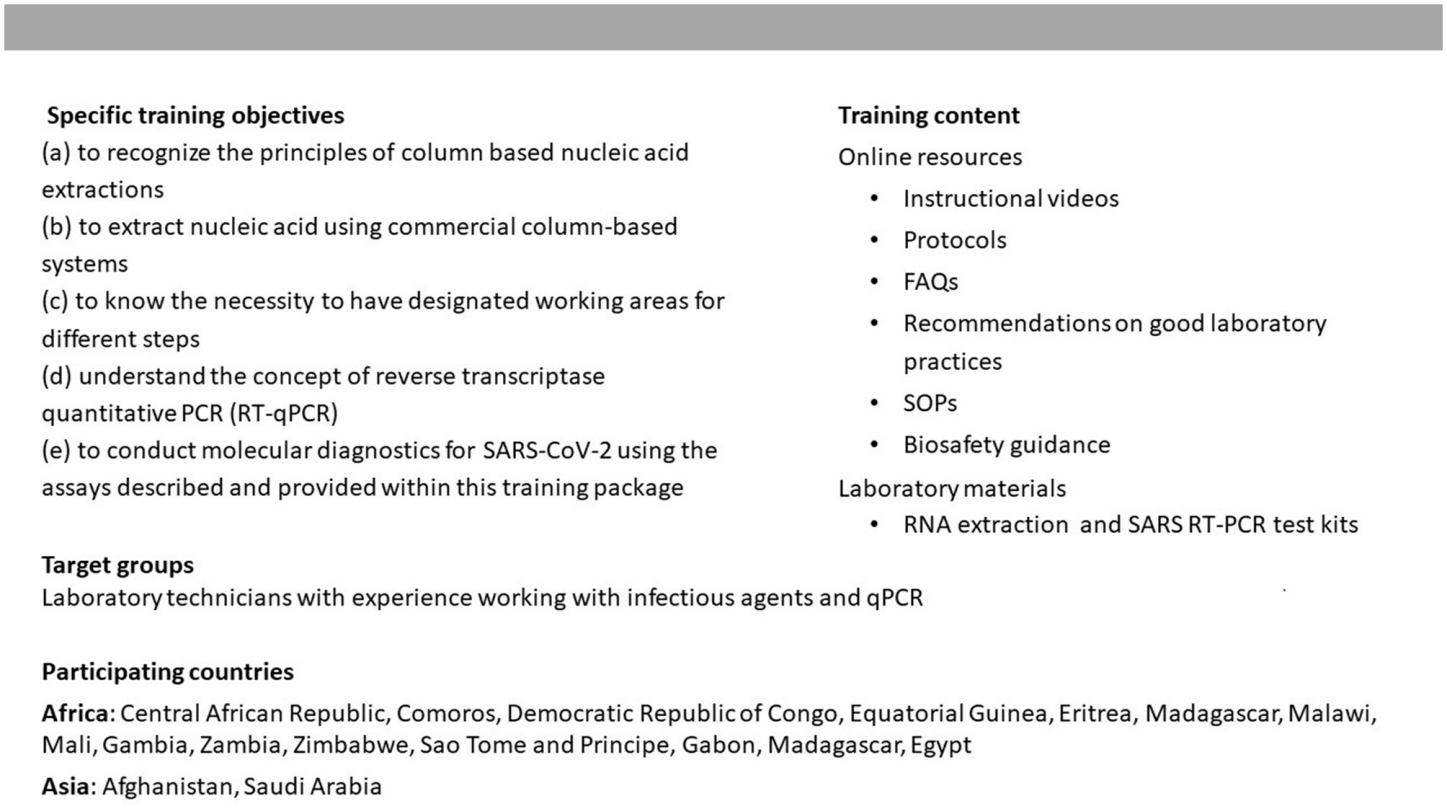
Characteristics of the online SARS-CoV-2 qPCR training.

The training was aimed at laboratory technicians with experience working with infectious agents and SARS-CoV-2 testing. The main objective of the training was to build participants' capacities in performing SARS-CoV-2 qPCR diagnostics. In addition, the training included instructions on good laboratory practice and on biosafety to refresh participants' knowledge of working with potentially contagious germs. For this purpose, ZIG 4 designed a training program that included video-based instructions, standard operating procedures (SOPs), and documents that detailed the technical aspects of the diagnostic methods. In addition, reagents and other diagnostic disposables were sent to 23 laboratories that were engaged in the COVID-19 response in RKI partner countries and had voiced an interest in participating in the training. All the training material was initially made available via an online platform (https://zenodo.org/record/4058349) in English, and French. Following the launch of the training, additional laboratories inquired about the training materials leading to additional translations to Russian and Spanish. ZIG4 held a live webinar to launch and present the English and French training materials. Following the launch, ZIG4 trainers were available to provide support through email or teleconference exchange as well as a weekly question and answer sessions over a period of 8 weeks. The training was largely developed and implemented via a top-down approach. End users were not engaged in the development and implementation of the training due to the immense time pressure at the beginning of the COVID-19 pandemic and the urgent need to quickly provide laboratory staff with a SARS-CoV2 diagnostic training that could be implemented in LMIC. While the pursuit of a top-down approach is comprehensible given these exceptional contextual circumstances, it does not represent best practice for developing and delivering capacity building interventions and runs the risk of the training not engaging with or meeting the needs of end users. Furthermore, the training was a one-time intervention. More information on the training is provided in Figure 1.

In this study, we analyse the perception of users of this online SARS-CoV-2 qPCR diagnostic training; compare the costs of the online vs. a face-to-face SARS-CoV-2 qPCR diagnostic training; and assess the potential of the online training to build health system resilience and local capacities in a public health emergency context. Drawing on this, we offer recommendations for improving online diagnostics training in public health emergencies.

## Methods

We designed a concurrent mixed-methodology study based on key informant interviews, interviewer-administered questionnaires, and document reviews.

### Analyzing the perception of users of an online SARS-CoV-2 PCR diagnostic training

Drawing on the literature on quality learning methods (14, 15) and following the criteria of the Development Assistance Committee (DAC) of the Organization for Economic Co-operation and Development (OECD) for evaluating public health interventions as well as a review of the content of the online training materials, a topic guide was developed for semi-structured interviews. Aimed at understanding the perception of the training participants and assessing the potential of the training to build health system resilience and local capacities, the topic guide covered the following categories: involvement in the training, planning and implementation, effectiveness, relevance, coherence, sustainability and scale up, and overall evaluation of the training. Table 1 provides examples to illustrate what kind of questions were asked in each category.

**Table 1:** Categories and exemplary questions of topic guide for key informant interviews.

**Category**	**Exemplary questions**
Involvement in the training	• What was your role in the PCR-CoV2 diagnostics training (design/planning/implementation)?
Planning and implementation	• Can you tell me about the process from designing the training through to implementation? • Which kind of learning methods did you base the training on?
Effectiveness	• Can you tell me how the training affected the daily work activities of those working in the labs?
Relevance	• How did the training fit within your organization's mandate?
Coherence	• How did the online training fit with the other training offers?
Sustainability and scale up	• In what kind of ways were you able to provide input/feedback on the training? • Was the training changed at some point after its planning, e.g., after site testing or implementation or when it was passed on to other partners?
Overall evaluation of the training	• What is your overall assessment of the training? • What could have been done better?

We sampled training participants for the key informant interviews as follows. First, we obtained from ZIG4 the list of all 23 laboratories in 17 partner countries (the full list of countries is shown in Figure 1) which participated in the SARS-CoV-2 qPCR online training. Training participants were invited to take part in the study through an email invitation to the director or contact of each participating laboratory. At least one person from each participating laboratory was invited to take part in the interview process. All contact persons were contacted at least two times for an interview. Contact persons that did not respond after two attempts were not contacted further. Six key informant interviews took place with individuals from six different countries based in Africa between November 2020 and January 2021 via the telephone or online in English or French. Each interview was audio recorded and detailed notes were taken and summarized.

### Comparative cost analysis of online vs. face-to-face SARS-CoV-2 PCR diagnostic training

We estimated (1) the economic costs (i.e., the costs of all resources consumed) associated with delivering the one-time online training to the 23 participating laboratories, (2) the economic costs of delivering the same training to the 23 participating laboratories had the training mode been face-to-face, and (3) the net costs of the one-time online vs. the one-time face-to-face training. The perspective of the cost analysis was that of RKI. To undertake the comparative cost analysis, we performed an interview-based survey of all RKI staff involved in the online SARS-CoV-2 qPCR diagnostic training and in face-to-face trainings prior to the implementation of travel restrictions, and a financial record review. Both the online and the face-to-face trainings were defined in terms of their component activities. For each activity we estimated resource consumption, specifically the (1) time of professionals, (2) consumables, (3) capital equipment, (4) office space, and (5) other resources (e.g., courier costs, custom clearance costs, travel costs) required to undertake the activity. We valued all resources at their unit prices and included value-added tax (VAT) where applicable. Capital equipment costs and office space costs were estimated at their equivalent annual costs, i.e., discounted at an annual rate of 3% over their expected lifetime (16). Table 2 provides a few examples of the types of questions that were asked to RKI staff in the interview-based survey to estimate the costs of the online SARS-CoV-2 qPCR training.

**Table 2:** Exemplary questions of the cost survey to RKI staff for the online SARS-CoV-2 qPCR training.

**Type of resource**	**Exemplary questions**
Time	• In total, how many hours did you spend designing, writing up materials (SOPs, FAQs, PPTs), filming and editing videos, revising materials and, if necessary, translating materials? • How much time did you spend in total in the Kick-off Webinar?
Equipment and consumables	• If you purchased or rented any laboratory or office equipment to provide training support (i.e., to be able to implement the kick-off webinar, Q&A sessions or other training support activity), please name the equipment; • If you purchased any laboratory or office consumables to provide the training support (for example, stationary), please name the consumables;
Transport	• What means of transport did you use when you traveled outside of your normal work commute to perform the activities required to prepare the training? • If you paid money out of pocket in transport fares when you traveled outside of your normal work commute to prepare the training, how much money did you pay in total (go and return)?

We estimated the total, activity, and resource-specific costs of the online and of the face-to-face training assuming that the face-to-face training would have been performed in 1 week (as had been done by RKI staff before the travel restrictions were implemented) and in four geographical locations (West Africa, East Africa, Southern Africa, and Middle East) as was deemed appropriate considering the geographic distribution of the 23 partner laboratories. We estimated attendance to each 1-week face-to-face training at 15 laboratory technicians per training. We further estimated the total, activity and resource-specific net costs of online vs. face-to-face training. Throughout, costs are expressed in euros at 2020 price levels.

Informed consent was obtained from all individuals participating in the study. Ethics approval for the study was obtained by the Charité Universitätsmedizin Ethics Review Committee (ID: EA1/346/20).

## Results

### Perception of the usefulness of online PCR diagnostic training and suggestions

The interview sample comprised of two interviewees working at a national reference laboratory, one in a research institute, one at public health institute, one in the Ministry of Health, and one interviewee in charge of developing, organizing and implementing the online training. Using a thematic analysis, three themes emerged from the data: respondents' assessment of the content and format of the training, the perceived impact of the training, and the reported factors perceived to influence the effect of the training.

#### Assessment of the content and format of the training

Interviewees assessed both the content and the format of the training. As of August 2, 2023, the course materials on the website had received 8,984 views and had been downloaded 2,958 times. Note that these figures are not necessarily indicative of the number of people who may have actually used the materials (for example, individuals may have made multiple downloads). With reference to the training content, the participants described that the course provided good and comprehensive information on up-to-date scientific knowledge and laboratory practice in PCR diagnostics. Respondents appreciated how the technical content went beyond molecular biology teaching and supported participants' understanding of molecular mechanisms and enhanced their ability to apply diagnostic methods in their daily work. Several participants noted how important it was for the course to include teaching on biosafety, especially for people working with potentially contagious samples for the first time. Most interviewees said that the content was well-adapted and suitable for the target audience and context. However, some interviewees highlighted how labor-intensive and time-consuming the methods were especially concerning the high demand and that participants with limited background knowledge had difficulties to implement them. The participants mentioned several points regarding the format of the course. First, they expressed that the dissemination had been adequate. Some participants suggested that connecting with other regional organizations, such as the African Society for Laboratory Medicine (ASLM), would have been useful to increase knowledge exchange and dissemination and could have helped to streamline the communication between laboratories and local authorities. Secondly, participants reviewed the online platform used for the training in a mixed way. While they highlighted that accessing the material was easy and they could attend the training in their own time due to the asynchronous nature of the course, they stressed the lack of interactive tools and reported that the cumbersome nature of the platform limited the learning experience. Participants referred to the Standard Operating Procedures (SOPs) and other training material as clear and concise, but some pointed out the limitations experienced due to the language barriers and other logistical problems, including limited internet connection, limitations of facilities that allowed internet access in which the training could be attended, delays in receiving the laboratory material and shortage of reagents. One respondent described the logistical challenges that restricted the number of laboratory personnel that was able to attend the training: “A big video conference room that would allow a large number of people to attend the training wasn't available […] so we couldn't train everyone at once. We had to break up the group”. Thirdly, the interviewees discussed several lost opportunities with reference to two-way communication and feedback between participants and trainers. Several participants were not aware of the option to communicate with the trainers via weekly Q&A sessions directly and bemoaned the lack of opportunities to directly discuss technical challenges with the trainers. In line with this, the organizers reported that no-one had participated in these sessions. From the trainers' perspective, the lack of interaction meant that they were unable to assess if the participants had understood and were able to apply the diagnostic methods.

#### Perceived impact of the training

With regard to the perceived impact of the training, the interviewees indicated multiple positive aspects of the course. Interviewees mentioned that the training had provided timely possibilities to learn new methods that were crucial in their diagnostic work during the COVID-19 pandemic, improved their technical laboratory skills and expanded them to new diagnostics methods, provided information on biosafety, and had helped them to incorporate good laboratory practices into their working routines. The participants noted that the course helped to build local laboratory capacities by allowing participants to act as trainers themselves and providing their colleagues with subsequent internal training and supervision. One respondent recalled internal trainings that had been conducted to scale down the training: “So we just picked a few people to attend the training and these would then teach the others in the lab. We can't all sit around the webinar, but we have a few selected that then teach the others in the laboratory”. In one particular instance, an interviewee underscored how the course had sparked the creation of a local community of learners, had facilitated internal discussions to solve problems, exchange knowledge, and support colleagues in developing technical skills. One participant even described that other local experts, such as WHO officials, had been part of these communities of learners. Interviewees also expressed how the course enhanced their bargaining position as a laboratory with their governments due to increased knowledge and capacity. In addition, interviewees mentioned that the training created opportunities to launch and build international collaborations and increase networks between local and international laboratories.

#### Factors perceived to influence the effect of the training

Several participants mentioned factors that they perceived as influencing the effect of the training. These included personal factors, such as motivation, previous knowledge, and a perceived need to improve their laboratory skills. Several interviewees highlighted that the fast implementation and the low cost of the online format had allowed them to quickly build skills that were crucial during, and beyond, the COVID-19 crisis. Several participants highlighted that the training had been available at the right time and shortly after the outbreak emerged. Yet, the delivery of the course during a high demand due to the pandemic also meant that training participants and supervisors had to weigh a high workload against their training needs. Interviewees further mentioned how the effect of the training highly depended on the local circumstances and the support that participants had received. One respondent stressed the need for support and implementation by laboratory managers in order to ensure impact: “The success of the training depends to some extent on […] managers who oversee the team taking the training and whether they ensure that the training contents are applied afterwards.” Similarly, some interviewees discussed how their supervisors supported them to take the training, therefore putting into practice what they had learned. They also mentioned the importance of available lab equipment and workload.

### Comparative cost analysis

**Figure 2:**
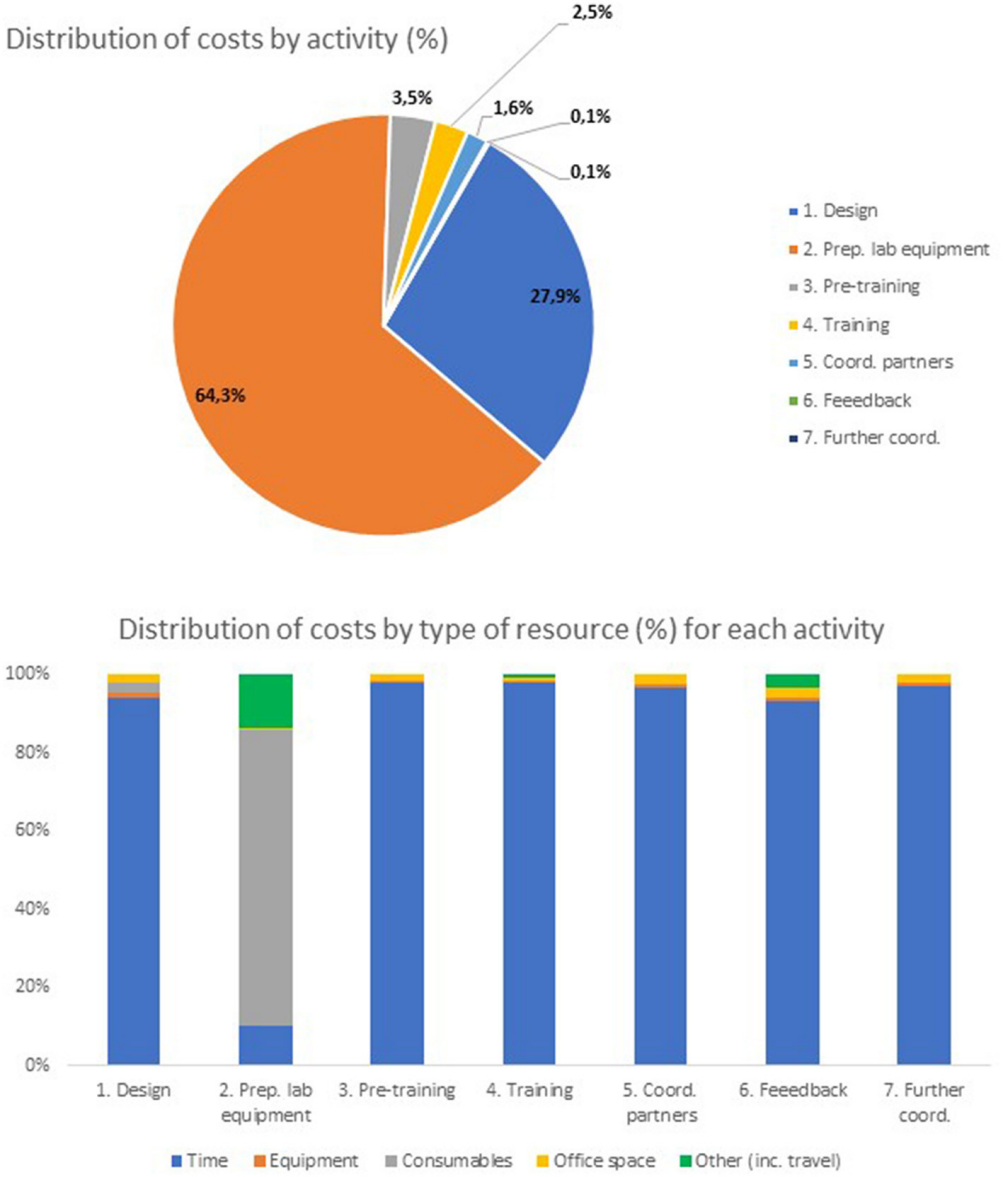
Distribution of costs of online SARS-CoV-2 qPCR training by activity and by type of resource.

Table 3 presents, from the perspective of RKI, the sequence of activities incurring in costs identified for both the online and the equivalent face-to-face trainings. Table 4 presents the total costs of the online SARS-CoV-2 PCR diagnostic training by activity. Figure 2 below shows the distribution of costs by activity (top panel) and, within each activity, the distribution of costs by type of resource (bottom panel).

**Table 3:** Sequence of activities: online and face-to-face SARS-CoV-2 qPCR trainings.

**Activities**
**1. Design of training materials**, including: writing up standard operating procedures (SOPs)/lectures/presentations/other documents/filming and editing videos/revising materials/translating materials
**2. Preparation of laboratory equipment and PCR kits for the training**, including: researching and purchasing the PCR test material and the relevant consumables/preparing the kits for shipment to the partner laboratories/organizing the shipments (i.e., performing the relevant administrative work to be able to make the shipping, including customs clearance)/making the shipments/following-up on the shipments until they reach their final destination
**3. Coordination with partners (pre-training)**, including: negotiating and establishing the final list of participating laboratories, coordinating with participating laboratories, laboratory staff and other partners the training activities, organizing the reception of training materials and PCR kits, ensuring the training materials and PCR kits were ready for the training
**4. Training and/or training support**, including: (1) for the online training, a kick-off webinar; weekly Q&A sessions; additional support activities; (2) for the face-to-face training, lectures, practicals and additional support activities
**5. Coordination with partners during the training** in order to facilitate the training
**6. Provision of feedback to participating labs or participants** regarding the training after the training
**7. Coordination with partners for further training**

**Table 4:** Costs of online SARS-CoV-2 qPCR training (%) by activity (2020 euros).

**Type of resource**	**Costs of online training (%) by activity**							
	**1. Design of training materials**	**2. Prep. of laboratory equipment and PCR kits**	**3. Coord. with partners (pre-training)**	**4. Training and/or training support**	**5. Coord. with partners (during training)**	**6. Feedback to participants or participant labs regarding the training**	**7. Coord. with partners for further training**	**Total (all activities)**
1. Time of staff	16,165 (65.1)	3,995 (16.1)	2,087 (8.4)	1,534 (6.2)	976 (3.9)	61 (0.2)	31 (0.1)	24,849 (100)
1.1. Work time	16,116 (65.0)	3,995 (16.1)	2,087 (8.4)	1,534 (6.2)	976 (3.9)	61 (0.2)	31 (0.1)	24,800 (100)
1.2. Travel time	49 (0.03)	0 (0)	0 (0)	0 (0)	0 (0)	0 (0)	0 (0)	49 (100)
2. Equipment	229 (74.9)	47 (15.2)	14 (4.4)	6 (2.1)	10 (3.1)	0.7 (0.2)	0.3 (0.1)	307 (100)
2.1. Laboratory equipment	51 (100)	0 (0)	0 (0)	0 (0)	0 (0)	0 (0)	0 (0)	51 (100)
2.2. Other equipment	179 (69.8)	47 (18.3)	14 (5.3)	6 (2.5)	10 (3.8)	1 (0.2)	0.2 (0.1)	256 (100)
3. Consumables	441 (1.4)	30,029 (98.6)	0 (0)	0 (0)	0 (0)	0 (0)	0 (0)	30,470 (100)
3.1. Laboratory consumables	387 (1.3)	30,029 (98.7)	0 (0)	0 (0)	0 (0)	0 (0)	0 (0)	30,416 (100)
3.2. Other consumables	54 (100)	0 (0)	0 (0)	0 (0)	0 (0)	0 (0)	0 (0)	54 (100)
4. Office space	380 (64.3)	129 (21.9)	36 (6.1)	17 (3)	26 (4.4)	2 (0.2)	1 (0.1)	591 (100)
5. Other	0 (0)	5,434 (99.7)	0 (0)	11 (0.2)	0 (0)	2 (0.1)	0 (0)	5,447 (100)
Total	17,215 (27.9)	39,635 (64.3)	2,136 (3.5)	1,568 (2.5)	1,011 (1.6)	66 (0.1)	32 (0.1)	61,644 (100)

From Table 4, the total cost of the online training (with 23 participating laboratories) was, from the perspective of RKI, 61,644 euros. From Table 4 and Figure 2 (top panel), the activity with the highest cost was preparation of laboratory equipment and PCR kits for the training (39,635 euros, 64.3% of the total cost). Most of the resource consumption for this activity (35,463 euros, 57.5% of the total online training cost) went to laboratory consumables and other costs (including shipping costs and custom clearance costs—see Figure 2, bottom panel) of the materials necessary to perform the qPCR tests. The activity with the second highest cost was design of training materials (17,215 euros, 27.9% of the total cost). Within this activity, most of the resource consumption was associated with the time of staff required to design the training materials, including the time spent producing videos for distance learning. In total, this cost amounted to 16,165 euros, about 26.2% of the total online training cost. The activity with the third highest cost was pre-training coordination with partners (2,136 euros, 3.5% of the total cost). The remaining activities consumed substantially fewer resources (see Figure 2, top panel).

**Figure 3:**
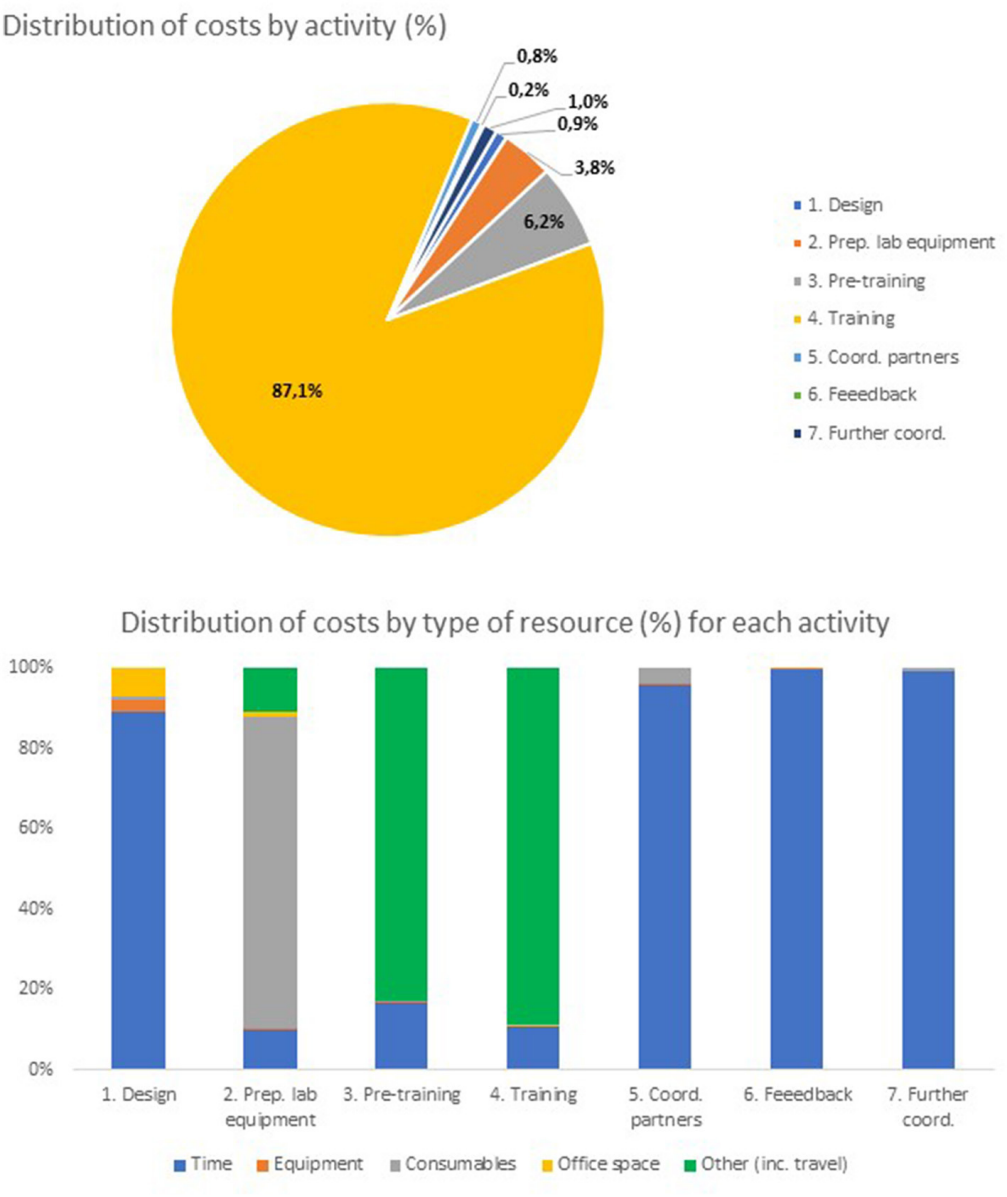
Distribution of costs of face-to-face SARS-CoV-2 qPCR training by activity/type of resource.

Table 5 below shows the total costs estimated for the equivalent face-to-face SARS-CoV-2 qPCR diagnostic training by activity. Figure 3 below shows the distribution of costs by activity (top panel) and, within each activity, the distribution of costs by type of resource (bottom panel).

**Table 5:** Costs of face-to-face SARS-CoV-2 qPCR training (%) by activity (2020 euros).

**Type of resource**	**Costs of face-to-face training (%) by activity**							
	**1. Design of training materials**	**2. Prep. of laboratory equipment and PCR kits**	**3. Coord. with partners (pre-training)**	**4. Training or training support**	**5. Coord. with partners (during training)**	**6. Feedback to participants or participant labs regarding the training**	**7. Coord. with partners for further training**	**Total (all activities)**
1. Time of staff	2,087 (5.8)	1,013 (2.8)	2,823 (7.8)	25,039 (69.1)	2,013 (5.6)	491 (1.4)	2,700 (7.5)	36,165 (100)
1.1. Work time	2,087 (8.2)	1,013 (4.0)	761 (3.0)	16,447 (64.5)	2,013 (7.9)	491 (1.9)	2,700 (10.5)	25,511 (100)
1.2. Travel time	0 (0)	0 (0)	4,124 (32.4)	8,592 (67.6)	0 (0)	0 (0)	0 (0)	12,716 (100)
2. Equipment	62 (28.0)	33 (15.0)	11 (4.8)	102 (45.9)	6 (2.7)	1 (0.7)	7 (2.9)	222 (100)
2.1. Laboratory equipment	0 (0)	0 (0)	0 (0)	76 (100)	0 (0)	0 (0)	0 (0)	76 (100)
2.2. Other equipment	62 (42.5)	33 (22.8)	11 (7.3)	26 (17.7)	6 (4.1)	1 (1)	7 (4.6)	146 (100)
3. Consumables	19 (0.2)	8,011 (94.1)	10 (0.1)	381 (4.5)	80 (0.9)	0 (0)	16 (0.2)	8,516 (100)
3.1. Laboratory consumables	0 (0)	6,886 (100)	0 (0)	0 (100)	0 (0)	0 (0)	0 (0)	6,886 (100)
3.2. Other consumables	19 (1.2)	1,125 (69)	10 (0.6)	381 (23.4)	80 (4.9)	0 (0)	16 (1)	1,630 (100)
4. Office space	168 (12.7)	92 (7.1)	11 (0.8)	1,048 (79.4)	0 (0)	0 (0)	0 (0)	1,319 (100)
5. Other	0 (0)	1,125 (0.5)	13,852 (6,3)	206,394 (93.2)	0 (0)	0 (0)	0 (0)	221,370 (100)
Total	2,335 (0.9)	10,273 (3.8)	16,706 (6.2)	232,963 (87.1)	2,099 (0.8)	492 (0.2)	2,723 (1)	267,592 (100)

From Table 5, the total economic costs to RKI of the face-to-face SARS-CoV-2 PCR diagnostic training equivalent to the online training was estimated at 267,592 euros. From Figure 3 (top panel), the activities with the highest costs were estimated to be training/training support (232,963 euros, 87.1% of all costs), pre-training coordination with partners (16,706 euros, 6.2% of all costs) and preparation of laboratory equipment and PCR kits (10,273 euros, 3.8% of all costs). From Figure 3 (bottom panel), time of staff is by far the most used resource in all activities except (i) preparation of laboratory equipment (where the costs of laboratory consumables are largely predominant), (ii) pre-training (where other costs, in particular the costs of traveling to coordinate the start of training with partners, constitute up to 13,852 euros, 83% of all costs for this activity) and (iii) training (where other costs, specifically the costs of travel and accommodation of trainers and trainees, amounted to 206,394 euros, 89% of all costs for this activity).

Table 6 shows the net costs of the online training with respect to the face-to-face training by activity and type of resource, with percentage changes included for the total columns and rows.

**Table 6:** Net costs of online vs. face-to-face SARS-CoV-2 qPCR training (2020 euros).

**Type of resource**	**Net costs of online vs. face-to-face training by activity**							
	**1. Design of training materials**	**2. Prep. of laboratory equipment and PCR kits**	**3. Coord. with partners (pre-training)**	**4. Training or training support**	**5. Coord. with partners (during training)**	**6. Feedback to participants or participant labs regarding the training**	**7. Coord. with partners for further training**	**Total (all activities)**
1. Time of staff	14,078	2,983	−736	−23,505	−1,037	−430	−2,670	−11,317 (−46%)
1.1. Work time	14,029	2,983	1,326	−14,913	−1,037	−430	−2,670	−712 (−3%)
1.2. Travel time	49	0	−4,124	−85,92	0	0	0	−12,667 (−25,800%)
2. Equipment	167	14	3	−95	4	−1	−6	85 (+28%)
2.1. Laboratory equipment	51	0	0	−76	0	0	0	−25 (−50%)
2.2. Other equipment	117	14	3	−19	4	−1	−6	110 (+43%)
3. Consumables	422	22,019	−10	−381	−80	0	−16	21,954 (+72%)
3.1. Laboratory consumables	387	23,143	0	0	0	0	0	23,530 (+77%)
3.2. Other consumables	35	−1,125	−10	−381	−80	0	−16	−1,576 (−2,897%)
4. Office space	212	37	25	−1,031	26	2	1	−728 (−123%)
5. Other	0	4,309	−13,852	−206,383	0	2	0	−215,923 (−3,964%)
Total	14,880 (+86%)	29,361 (+74%)	−14,570 (−682%)	−231,394 (−14753%)	−1,088 (−108%)	−427 (−647%)	−2,691 (−8,505%)	−205,928 (−334%)

From Table 6, the total net cost of the online SARS-CoV-2 PCR diagnostic training is estimated to be −205,928 euros, a full 334% cheaper than the equivalent face-to-face training. By activity, the largest savings occur in the training and/or training support (a net cost of −231,394 euros) and in the pre-training coordination with partners (a net cost of −14,570 euros). By type of resource, the largest savings occur in other costs (−215,923 euros, mostly due to savings in travel and accommodation of trainers and trainees) and in time of staff (−11,317 euros, mostly due to no face-to-face training requirements for the online modality). Conversely, by activity online training has a large positive cost in both the preparation of training materials and qPCR kits for training (+29,361 euros, mostly associated with procuring and sending consumables required for the PCR tests) and in the design of training materials (+14,880 euros, related to the time required for preparing the videos and other online training materials).

## Discussion

This paper provides an analysis of the perceived usefulness and the costs of a one-time online training on SARS-CoV-2 qPCR diagnostics in low- and middle-income settings during the COVID-19 pandemic. The analysis shows that online diagnostics training can provide an added value during a crisis and can have a positive effect in terms of allowing laboratory personnel to establish new diagnostic procedures and work with new technologies, and thus help to contain virus transmission and support emergency response. In particular, the analysis stresses how such training can provide good and comprehensive information on up-to-date scientific knowledge and laboratory practice in qPCR diagnostics, biosafety and good laboratory practice. The analysis also shows that, compared to face-to-face training, online SARS-CoV-2 qPCR diagnostic training can incur in substantial cost savings for RKI, the provider of this training.

The total cost of the one-time SARS-CoV-2 PCR diagnostic online training (with 23 participating laboratories) was, from the perspective of RKI, 61,644 euros. The total economic costs to RKI of the face-to-face SARS-CoV-2 PCR diagnostic training equivalent to the online training was estimated at 267,592 euros. Importantly, as the training was a one-time intervention, the cost calculations did not factor in the costs of any follow-up support to SARS-CoV-2 qPCR diagnostic training to the participating laboratories. In this sense, the costs of sustaining the training in the longer term, which would be substantial, are not included. In terms of the comparative cost analysis, from the perspective of RKI the one-time online SARS-CoV-2 qPCR training modality incurred, at scale, in very substantial overall cost savings compared to the equivalent face-to-face training. While the costs of the online training were higher than those of the equivalent face-to-face training in terms of mostly the staff time and the consumables required for, respectively, preparing the training materials and ensuring the 23 partner laboratories had access to the qPCR diagnostic materials and samples, these increased costs were more than compensated by very large savings in terms of travel and accommodation costs as well as face-to-face staff training time. Overall, the online training was more than three times (334%) cheaper than the equivalent face-to-face training.

We found no study addressing the cost-effectiveness or cost-savings associated with the type of non-traditional laboratory training intervention described in this paper, in which laboratories receive video training materials and laboratory consumables through the post, as well as online support in the form of webinars and Q&A sessions, while they use their own laboratory equipment to perform the learning experience. Some studies have explored the impact on the costs of e-learning, such as the review by Frehywot et al. (17). In their review, the authors discuss how e-learning and other distance learning techniques can affect economies of scale: once the materials are produced, the costs per participant are reduced when the training programmes are provided to large and repeated classes of learners (17). Sissine et al. found similar results for a blended (hybrid) e-learning training programme for community health workers in LMIC (18). They found that implementing the blended e-learning programme at scale (i.e., to 100,000 community health workers) could lead to a 42% reduction in costs compared to face-to-face training (18). We envisage substantial economies of scale in the SARS-CoV-2 qPCR online training presented in this study.

While highlighting the advantages of online training, our study also highlights contextual limitations of the SARS-CoV-2 qPCR diagnostic online training. The main contextual limitation that we identified was that of infrastructure difficulties, such as limited access to the internet for video visualization, limited space to undertake the training, delays in reception of equipment, and shortage of diagnostic reagents. Lack of internet access has been identified as a main weakness of online training (19), and lack of reliable internet performance is problematic in many parts of Africa (20). Poor laboratory resources/infrastructure and logistics constraints are a known challenge to COVID-19 testing in African countries (21). In addition, in Africa, global shortages limited access to laboratory reagents during the pandemic (22). While RKI trainers made sure that enough equipment and reagents were sent to participating laboratories for training purposes, there were shipping delays which were likely affected by the travel restrictions and other supply chain disruptions that were present in early 2020, when the online training was conducted.

We also identified several areas that could be improved in order to make the SARS-CoV-2 qPCR diagnostic online training more useful and align it with local needs and pandemic requirements. First, there was little direct engagement between trainers and course participants throughout the training. As outlined above, due to the immense time pressure at the beginning of the COVID-19 pandemic and the urgent need to quickly provide laboratory staff with a SARS-CoV2 qPCR diagnostic training that could be implemented in LMIC, end users were neither involved in the design, testing for understandability, cultural appropriateness or utility, nor in the testing of the translations of the materials or in the adaptation of translated materials to their specific cultural context. Such involvement would be an opportunity for improving the online training materials. Further, participants were not involved in deciding the format of interaction between trainers and end users once the latter had received the training materials. While trainers were available via e-mail, a kick-off webinar and weekly Q&A sessions to exchange with course participants, interactions were extremely limited. Several of the participants commented that they were largely unaware of these options for exchange with trainers. Limited attendance to (and attention during) online sessions and communication problems between trainers and participants are known limitations of online training (19). However, the limited exchange during the SARS-CoV-2 qPCR diagnostic training had the added effect that trainers did not receive feedback that could have improved the training materials or training format.

In order to identify opportunities to improve the engagement of course participants in the development and implementation of a SARS-CoV-2 qPCR diagnostic online training, is it useful to understand the drivers of end user engagement in online trainings. A recent systematic literature review and meta-analysis (23) explored the factors affecting user engagement in online professional training programmes, i.e., in a similar context to that of the SARS-CoV-2 diagnostic online training. In the meta-analysis, the review identified learner's technological self-efficacy (one's belief in one's ability to perform a sophisticated task such as using a computer), perception of course usefulness, ease of use of the online platform, environmental support (e.g., support from peers or other influential individuals) and facilitating situational influences (e.g., no time pressure, availability of resources, availability of assistance) as positively affecting emotional engagement (the learner's satisfaction with the online training) (23). Similar factors positively affected cognitive engagement (the learner's efforts to engage in online learning) (23). The meta-analysis further identified facilitating situational influences as positively affecting behavioral engagement (the learner's actions on the online platform, such as time spent participating in the online training or course completion) (23). In light of the results from this review and the themes we identified in our study, we propose three axes along which to involve course participants and more generally participating laboratories. First, usability testing of the course content and format with prospective course participants. Such testing will allow to make changes to the course content and format based on participant's feedback that ensure course participants can accurately and completely finalize the training with a limited level of effort and a high level of satisfaction. We hypothesize that usability testing will reveal the importance of our second proposed axis to involve participants, namely facilitating situational influences such as live interactions with trainers and other course participants (e.g., via webinars or online conferences) for discussing content, problems and practical experiences as has been previously reported by laboratory students (24). We also hypothesize that usability testing will highlight the importance of cultural adaptation of materials to the local context (25). Third, fostering environmental support via, for example, actively engaging laboratory managers in supporting the online training initiative and providing guidance toward using the skills learned by course participants after the initial training had ended.

Another opportunity for improvement lies in the establishment of communities of learners to increase knowledge sharing and dissemination, which was limited likely at least in part due to the online platform not allowing course participants and participating laboratories to interact with each other. Despite this, a small number of recipients reported that they had applied a train-the-trainer approach or built learning communities with colleagues and laboratory personnel out of their own initiative in their local context and even expanded their networks to experts in related areas. Finally, another opportunity for improvement would be incorporating a longer-term perspective to the online training. As mentioned previously, the SARS-CoV-2 qPCR diagnostic online training was a one-time intervention. It was motivated by the urgent need to support laboratories in partner countries with SARS-CoV-2 qPCR diagnostics training as quickly as possible and did not have a sustainability component built into it.

Online trainings have some advantages over face-to-face trainings. For example, the flexibility of training schedules, opportunities for incorporating multimedia resources (26) and, as previously mentioned, the opportunity of live interactions via webinars or online conferences (24). However, while face-to-face trainings tie students to specific schedules and higher costs (19, 26), they also have advantages over online trainings. Important benefits of face-to-face trainings are personal interaction, including the accessibility of trainers, student-trainer relationships, opportunities for discussions and face-to-face interactions (26), as well as the resulting familiarity with, and trust in, fellow students and trainers (27). Increased interaction during face-to-face training can lead to a higher likelihood of knowledge exchange, mutual learning and networking, which can be particularly crucial for participants who otherwise would have few opportunities to engage with peers and more experienced colleagues.

A recent systematic review comparing the strengths and weaknesses of non-traditional, online, remote and distance laboratory experiences with that of face-to-face laboratory experiences (28) suggests that a well-designed non-traditional laboratory learning experience can be as effective as a face-to-face one. Specifically, the authors discuss course features which increase the success of such learning experiences, such as: active, visible and intentional engagement of trainers with students (29); instructional design focused on developing students' skills in self-regulated learning; and a good ability to regulate time, study environment and effort on the side of the students (28). The authors also suggest guidance for inquiry as a powerful pedagogical approach, including performance dashboards, prompts, and process constraints (28, 30). Additional elements promoting success of online laboratory environments include an online learning community which allows for collaboration between peers (28, 31–33) and a well-organized calendar for the course (34).

One important consideration with regard to online laboratory trainings is the potential impact of developing hybrid or blended training approaches. In fact, hybrid training approaches have been successfully developed during the COVID-19 pandemic. For example, in order to overcome gathering restrictions, in the Spring of 2020 researchers at the Department of Chemical Engineering in Qatar University (35) developed a hybrid approach to laboratory training. This approach combined (1) filmed theoretical classes on a whiteboard in the corresponding lab room to represent as closely as possible face-to-face teaching, (2) filmed instructions regarding how to use the relevant laboratory equipment, also from the lab room with the same purpose, and, crucially (3) once students had watched the filmed material, online lab classes with in-depth discussions regarding the filmed material and problem-solving tutorials (35). This approach resulted in effective learning of the course objectives by the cohort of students taking this course. Given that real hybrid diagnostic trainings can be difficult to implement during public health emergencies due to the risk of infection and related preventive measures, an alternative might be to provide regular, face-to-face training to establish solid expertise on laboratory procedures and diagnostics and build communities of learners in LMIC. These trainings and the networks that are developed in this way could then be easily complemented with intensive online training and interactive sessions to exchange information during public health emergencies. Such approaches might not only help to build expertise, but also connect those working on laboratory diagnostics in LMIC, support them in conducting their tasks effectively, and empower them to fulfill their roles within public health systems.

Our study has several limitations. First, we limited the cost analysis to the perspective of RKI rather than the perspective of all participants (i.e., both RKI and related labs). We believe that widening the perspective would have demonstrated further cost savings associated with online training. We initially included the partner laboratories and additional partner institutions in the costing study, but due to low study participation we did not have sufficient data on these partners to estimate their costs. Second, the response rate of those who were approached for an in-depth interview was low. This was due to the evaluation not being built as a core component into the design of the online training and to potential respondents being even busier than usual as the study was conducted in the middle of the COVID-19 pandemic. Despite making immense efforts to reach course participants, we encountered particularly low response rates to a survey that we had planned to conduct to assess the effectiveness of the training among participants and were therefore unable to analyse participants' own views on the training. These limitations highlight the importance of planning and designing evaluations alongside the design of capacity building activities, including online training courses.

## Conclusion

An online training was developed and implemented to support SARS-CoV-2 qPCR diagnostics in LMIC during the COVID-19 pandemic. The training was perceived as useful by recipients, notably enabling staff tasked with conducting diagnostics to follow good laboratory practice and implement novel laboratory procedures. In addition, it incurred in important cost savings compared to the equivalent face-to-face training. With view to future pandemics and in order to strengthen pandemic response and health system resilience, it is important that diagnostic training is designed and delivered according to the current state-of-the-art. This includes the pursuit of a complementary approach which combines online and offline formats. It is also crucial that online training comprises interactive features in order to build communities of learners among those involved in diagnostics, facilitate exchange of information, and thus better unlock the expertise and potential that exists among those working at the basis to fight public health emergencies.

## Data availability statement

The raw data supporting the conclusions of this article will be made available by the authors, without undue reservation.

## Ethics statement

The studies involving humans were approved by Charité Universitätsmedizin Ethics Review Committee (ID: EA1/346/20). The studies were conducted in accordance with the local legislation and institutional requirements. The participants provided their written informed consent to participate in this study.

## Author contributions

HW, FP-M, BG, FC, and CE designed the study. HW, FP-M, BG, FC, SA, TB, and CE collected the data. HW, FP-M, BG, FC, EL, and EM-M analyzed the data. HW and FP-M drafted the manuscript with substantial input from BG, EL, TB, EM-M, SA-A, and CE. All authors critically revised the manuscript and substantially contributed to the final draft. All authors read and approved the final manuscript.
